# Gaps and opportunities: measuring the key population cascade through surveys and services to guide the HIV response

**DOI:** 10.1002/jia2.25119

**Published:** 2018-07-22

**Authors:** Avi Joseph Hakim, Virginia MacDonald, Wolfgang Hladik, Jinkou Zhao, Janet Burnett, Keith Sabin, Dimitri Prybylski, Jesus Maria Garcia Calleja

**Affiliations:** ^1^ Division of Global HIV and Tuberculosis US Centers for Disease Control and Prevention Atlanta GA USA; ^2^ World Health Organization Geneva Switzerland; ^3^ The Global Fund to Fight AIDS, Tuberculosis and Malaria Geneva Switzerland; ^4^ Division of HIV/AIDS Prevention US Centers for Disease Control and Prevention Atlanta GA USA; ^5^ United Nations Joint Programme for HIV/AIDS Geneva Switzerland

**Keywords:** Key populations, 90‐90‐90 cascade, surveillance, surveys, programme monitoring

## Abstract

**Introduction:**

The UNAIDS 90‐90‐90 targets to diagnose 90% of people living with HIV, put 90% of them on treatment, and for 90% of them to have suppressed viral load have focused the international HIV response on the goal of eliminating HIV by 2030. They are also a constructive tool for measuring progress toward reaching this goal but their utility is dependent upon data availability. Though more than 25% of new infections are among key populations (KP)‐ sex workers, men who have sex with men, transgender people, people who inject drugs, and prisoners‐ and their sex partners, there is a dearth of treatment cascade data for KP. We assess the availability of cascade data and review the opportunities offered by biobehavioral and programme data to inform the HIV response.

**Discussion:**

The emphasis on the collection of treatment cascade data among the general population in higher prevalence countries has not led to a similar increase in the availability of cascade data for KP. The limited data available for KP highlight large gaps in service uptake across the cascade, particularly in the first 90, awareness of HIV status. Biobehavioral surveys (BBS), with linked population size estimation, provide population‐based data on the treatment cascade and should be conducted every two to three years in locations with services for KP. With the inclusion of viral load testing, these surveys are able to monitor the entire treatment cascade among KP regardless of whether these populations access HIV services targeting the general population or KP. BBS also reach people accessing services and those who do not, thereby providing a unique opportunity to learn about barriers to service uptake including stigma and discrimination. At the same time high‐quality programme data can play a complementary role in identifying missed opportunities that can be addressed in real‐time.

**Conclusions:**

Data are more important than ever for guiding the HIV response toward reaching 90‐90‐90 targets and eliminating HIV, particularly in the face of decreased funding for HIV and specifically for KP. Timely high‐quality BBS data can be triangulated with high‐quality programme data to provide a comprehensive picture of the epidemic response for KP.

## 1 Introduction

UNAIDS 90‐90‐90 targets provide a valuable framework to guide the HIV response and monitor progress towards ending the epidemic 1. These targets of having 90% of people living with HIV (PLHIV) aware of their infection, with 90% of them on antiretroviral therapy (ART), and 90% of them with suppressed viral load were conceived so that they could be informed through programme or survey data 2. Though programme data exist about the number of HIV tests conducted and the number of people on ART, estimates of ART coverage are largely based on models such as Spectrum, and until recently, little has been known about population viral suppression 3. However, the international embrace of the 90‐90‐90 targets has spurred surveys to collect data towards these indicators 4, 5. It has also led to a recognition that the key populations treatment cascade should be expanded to include outreach prevention and testing services as this is where many key population members first engage with the health system 6, 7.

In order to elucidate progress toward UNAIDS targets, the US President's Emergency Plan for AIDS Relief (PEPFAR) is assessing national and sub‐national 90‐90‐90 cascades among the general population in more than one dozen high HIV prevalence countries through the Population‐based HIV Impact Assessment (PHIA) 8. These surveys are seen as the best available method for nationally representative estimates of progress toward 90‐90‐90 targets. Results from Zimbabwe, Malawi and Zambia have highlighted the need to focus efforts on diagnosing infections because once diagnosed, countries are making good progress at linking and retaining people on antiretroviral treatment and suppressing viral load 9, 10. Data from Swaziland have confirmed the potential of combination prevention efforts and increasing treatment coverage in reducing HIV incidence 11, 12. However, while population‐based national and sub‐national 90‐90‐90 cascade data are quickly becoming available for the general population in higher prevalence countries, no population‐based surveys provide cascade data for key populations—sex workers (SW), men who have sex with men (MSM), transgender people (TP), and people who inject drugs (PWID). The lack of survey‐derived cascade data for key populations is striking missed opportunity given that a larger number of biobehavioral surveys (BBS) have been conducted among key populations than among the general population 13, 14, 15, 16, 17, 18.

In the absence of BBS data, key population programme data are often insufficient to fill data gaps about the cascade. These data are limited by the challenges of tracking individuals from community‐based interventions to health facilities, and from facility to facility, particularly if that facility does not utilize key population unique client codes 19, 20. In some settings, people may not feel comfortable reporting their sexual or drug injecting practices or identities and in others it may not be safe to collect such data due to stigma and discrimination. This lack of data impedes the effective targeting of services by population, location, and intervention need, and measuring of progress toward epidemic control. We conducted a literature search in PubMed and reviewed reports for PEPFAR and Global Fund‐supported surveys to identify surveys reporting at least two elements of the 90‐90‐90 cascade. We compare general population and key population cascades and their availability; and review the opportunities offered by BBS and programme data as well as their limitations.

## 2 Discussion

### 2.1 A tale of two responses: progress towards general population and key population epidemic control

The immense progress combating HIV in the general population, evidenced by PHIA results, has been rightly celebrated but simultaneously underscores how far there is to go with key populations. It is well established that the risk of HIV acquisition is far higher among key populations than the general population, that HIV prevalence is higher among key populations, and that key populations access to HIV services is low 21, 22, 23, 24. Even within the same country, recent national population‐based surveys (e.g. PHIA and Kenya AIDS Indicator Survey) and population‐based BBS among key populations put in stark contrast the disparities in the 90‐90‐90 cascades between general population and key population members in the same country. They further illustrate the low coverage among key populations or the absence of key population cascade data in other countries (Table 1). Of great importance is that no cascade data exist for transgender people apart from in combination with MSM and no publicly available data exist for prisoners. As BBS should be conducted in locations with key population services and populations of sufficient size for sampling, the number of sites contributing to key population estimates are indicated below 5.

**Table 1:** General population and key population 90‐90‐90 cascades

Country	Population	Sites	Year of data collection	First 90: self‐reported diagnosed	Second 90: self‐reported on ART of those diagnosed	Third 90: virally suppressed, of those on ART
Cameroon 25	FSW	5	2015 to 2016	52%	81%	a
Cameroon 25	MSM/TP	5	2015 to 2016	42%	63%	a
India 26	MSM	12	2012 to 2013	30%	53%	63%
India 26	PWID	15	2012 to 2013	41%	44%	83%
Kenya 27	General population	National	2012	62.4%	71.9%	79.8%
Malawi 10	General population	National	2015 to 2016	72.7%	89.6%	91.2%
Mozambique 28	General population	National	2015	34.3%	77.3%	a
Mozambique 29	FSW	3	2011 to 2012	22.3%	52.5%	a
Mozambique 29	MSM	3	2011	8.8%	39.8%	a
Mozambique 29	PWID	2	2014	63.2%	44.9%	a
Papua New Guinea 15	FSW	1	2016	38.9%	84.4%	54.6%
South Africa 30	General population	National	2012	37.8% male/55.0% female	25.7% male/34.7% female	a
South Africa 31	FSW	1	2014 to 2015	82%	48%	a
Swaziland 32	General population	National	2016 to 2017	84.7%	87.4%	91.9%
Uganda 14	FSW	1	2012	37.5%	67.7%	51.6%
Uganda 13	MSM	1	2012 to 2013	20.2%	75.0%	58.3%
Zambia 10	General Population	National	2015 to 2016	66.0%	85.0%	89.3%
Zimbabwe 9	General Population	National	2015 to 2016	72.9%	86.8%	86.5%
Zimbabwe 16	FSW	14	2013	64.0%	67.7%	77.8%

a Data not available.

Only in Malawi and South Africa is the proportion of PLHIV self‐reporting being aware of their HIV infection higher in a key population group (female sex workers, FSW, in this case) than the general population, though the South African key population survey occurred two years after the general population survey during which time services may have been expanded. Another explanation is that the FSW surveys were conducted in urban areas while the general population data represents the entire country, and HIV testing access, and therefore awareness, may be higher in urban than rural areas.

The limited BBS viral load in Table 1 may reflect the availability of viral load testing. The scale‐up of viral load testing for treatment monitoring in many resource‐limited settings will aid in estimating viral load measures in surveys. Where access to viral load testing is limited, investigators can send dried blood spots or plasma serum to other laboratories (e.g. in the capital or to another country) for testing while making an effort to return viral load results to survey participants.

Similar to the general population, the largest gap in 90‐90‐90 key population cascades above is among the proportion of people self‐reported to be living with HIV and aware of their infection. Where data exist, self‐reported awareness of HIV infection is lowest among MSM. While linkage to treatment is comparable between populations, it is still lower among key populations, and viral suppression data suggest that treatment adherence or retention may also be lower among key populations compared to the general population, signifying that all three steps in the key population cascade need attention, with priority on diagnosis.

Structural factors such as continued discrimination and criminalization of key populations impede access to health services and willingness to conduct surveys. Barriers to timely BBS include political will and funding as services are prioritized over strategic information to guide them, and the general population over key populations.

The representativeness and timeliness of data are extremely important for maximizing their utility. The data in Table 1 come from respondent‐driven sampling surveys for key populations and nationally representative cluster‐based household surveys for the general population, both of which are able to provide representative data about the target population. Though other data may exist, they are of lesser quality (i.e. utilized non‐probability probability sampling methods or were restricted to a key population sub‐group) and therefore not included. Table 1 reveals that key population BBS may not be conducted with sufficient frequency (i.e. every two to three years), and the publication dates of the data presented suggest that results may take a considerable amount of time to be released 5. Survey‐derived treatment cascades do not exist for key populations in all other resource‐constrained countries.

At a basic level, many southern African countries have no BBS data at all on men who have sex with men. In addition, Table 1 illustrates that where data do exist, data on the general population are generally more recent than on key populations. Key populations can play a critical role advocating for data collection. Kenya conducted BBS among key populations and a national general population survey in 2012 but only measured the cascade in the general population survey 33. While another national survey will be conducted in 2018, no survey was planned amongst female sex workers, men who have sex with men, or transgender people until recent advocacy efforts by civil society resulted in the allocation of resources for such surveys.

### 2.2 A tale of two cascades: population‐based and programme‐based

Population‐based surveys in the form of BBS are an essential part of key population surveillance and should be conducted every two to three years to measure changes in the epidemic and the impact of the response 2, 5. BBS have many advantages. Foremost is their ability to obtain data on people regardless of whether they are accessing HIV services, allowing the development of representative estimates of service coverage, including outreach and prevention services such as pre‐exposure prophylaxis, and 90‐90‐90 cascades for the survey location 2. By asking questions of participants about non‐engagement in services, BBS allow an understanding of the characteristics and reasons why people are not testing for HIV, not on treatment, or not virally suppressed, information that is essential for increasing HIV service coverage. Such reasons may include perceived or experienced stigma, cost, not knowing where to test, or feeling not at risk 13, 34, 35. BBS can also facilitate the production of population size estimates. They are often challenged, however, by the time required for planning, implementation, data analysis, and report writing, as well as budget constraints. Structural factors such as stigma and criminalization may also impede participation in BBS. This can be assessed through formative assessment and non‐response interviews during BBS, and mitigated by early engagement of key populations in survey planning.

It may be tempting to use data from general population surveys in place of BBS data to characterize the HIV epidemic; however, such efforts are fraught with many challenges and the surveys are only conducted in a subset of countries. Key populations may be less likely to be sampled in household surveys due to homelessness, or informal or clandestine living arrangements. Many ministries of health are reluctant to include questions about same‐sex sexual activity, transgender people, or even the gender of sex partners in household surveys, particularly when these are criminalized. Where key populations are sampled and questions about their practices and identities are included in a survey, they may not disclose them to interviewers. Taken together, these factors may bias key population cascades obtained in general population surveys. Finally, individual surveys may not have sufficient power to disaggregate data by key population. As surveys such as PHIA are powered for national incidence estimation and subnational viral load suppression for the general population, they lack the power to estimate the same for key populations who make up a small proportion of the population. General population surveys do, however, offer a unique opportunity to estimate national and subnational key population size through the use of the network scale‐up method. Such estimates may facilitate the expression of key population cascades when combined with high quality programme data.

Whereas BBS reach people accessing services and those who do not, programme data only describes those accessing services. In addition, key population programme data only describe those key population members who access services at key population‐friendly facilities that disaggregate data by population rather than all key population members living with HIV (KPLHIV) who access services, leaving an incomplete picture of KPLHIV who access services. Stigma continues to hinder uptake of services by key populations and disclosure of key population practices and identities among those who do access them, resulting in low programme coverage and consequently, inadequate data for drawing conclusions about the population 36, 37, 38, 39, 40. And in some countries, the provision of services to key populations has been barred, effectively excluding the possibility of using programme data to inform cascades 41. BBS can help highlight the detrimental effects of such policies and structural barriers. They can further assess mental health measures and the mediating role between exposure to stigma, violence, and discrimination, and practices, identities, and service uptake. The 2017 WHO Biobehavioral Survey Guidelines for Populations at Risk for HIV offers questions for investigators to include in order to measure these and other important topics, including shame and social cohesion 5.

High‐quality programme or service data that are individualized and deduplicated can provide important information about the number and sub‐groups of people accessing services and their ART outcomes 42. This can help service providers monitor interventions to verify that they are efficiently reaching the right people in the right places and that interventions are functioning as planned. When programme data reveal that the number of new people reached with outreach services decreases over time, when the testing yield declines, or when patients do not collect medicine, providers can respond in real time to changes in the population and their needs. Short surveys using audio‐computer assisted personal interviews can also be integrated into routine services to obtain behavioural data on individuals accessing services. Such surveys have been used at a testing facility for men who have sex with men in Uganda (e.g. the Know Your Sero‐Status, KYSS, survey) as well as at one of Uganda's largest general population testing facilities, Mildmay Clinic. A unique client code can be used to track people and their practices and serostatus over time. These data should not be used to replace BBS as they only represent people already accessing services. Furthermore, sentinel sites are ill‐suited for key population surveillance because their very nature, for instance as an sexually transmitted infections clinic, make them associated with service uptake and HIV, and the resulting data are biased.

Despite these advantages, data quality can vary widely and routine programme data only provide information about service uptake and health status. Detailed behavioural data that can inform service delivery generally are not collected. Tracking individuals, and linkage to and retention on treatment can also be challenging. Double counting of individuals accessing services at the same site, or at multiple sites or providers may inflate the reach of services or skew testing yield. The generation of unique client codes, as many key population services have done, may improve data quality but may also create challenges when trying to assess the number of key population members accessing services at key population‐friendly or general population services that do not use these unique identifiers or collect information about key population status. This is further limited where stigma or fear lead key population members not to disclose their defining risk practice or identity 43, 44, 45, 46. Noting the significance of confidentiality and security issues for key populations, WHO does not recommend the inclusion of key population groups on patient monitoring records 47. In addition, where it may seem that people diagnosed with HIV are not linked to treatment or that people on ART have defaulted, it may also be the case that individuals chose to seek services elsewhere 48, 49, 50. These silent transfers may prompt policy makers and service providers to target resources at a problem that may not exist. In addition, without a robust population size estimate, it is nearly impossible to estimate outreach coverage, the starting point of the key population cascade.

The triangulation of both BBS and programme data has great potential to guide service providers and policy makers in the epidemic response 51, 52, 53. Together, these data, collected using different methods and data sources, may be able to provide a comprehensive picture of the epidemic response, with programme monitoring revealing trends in service provision and missed opportunities, and BBS data on the reach and impact of services. For instance, programme data showing high loss to follow up and BBS data showing high ART coverage and viral suppression, may reveal that people are simply changing service providers, possibly even to general population sites, thereby suggesting that fewer resources are needed to identify and reengage those who were thought to be lost to follow up and retain others. The higher than expected viral suppression may also suggest a reduction in HIV incidence. While triangulation exercises should be updated regularly as new data become available, they are not common and the production of a report or publication is even less common.

It is imperative that key population members play a meaningful role in the collection and use of data about and for them. Formative assessments provide key populations with an important role in informing BBS. They should also be included as collaborators and investigators, and where appropriate, data collectors. In Papua New Guinea, survey results were shared with key population members ahead of public release and their feedback and recommendations were incorporated into survey reports, including in the form of statements from key population organizations 54, 55. Such engagement enhances a sense of ownership of the results by these groups and their ability to use the data for advocacy.

## 3 Conclusions

The HIV treatment cascade is a valuable tool for measuring progress toward epidemic control and when measured through BBS, elucidating barriers, structural and otherwise, to service utilization. While there are many gaps in measuring the cascade, there are also many opportunities for data collection and use. Though BBS of key populations are recommended to be conducted every two to three years, in practice they occur less frequently or not at all 5. Meanwhile, their importance for monitoring the epidemic will increase as countries get closer to reaching 95‐95‐95.

Many efficiencies can be found to expedite data collection and facilitate timely data availability and use, in turn decreasing costs of data collection and service provision. These efficiencies include starting surveys with more seeds, utilizing audio computer‐assisted self‐interviews that require fewer staff and decrease bias, and developing analytic programs during data collection 56, 57, 58. Incorporating BBS into national surveillance strategies and making them routine activities implemented every two to three years can help enhance the timeliness, standardization, quality, and utility of data. Following the example of the ARISTOTLE study which showed the cost effectiveness of their survey and intervention, other surveys are now beginning to assess the cost effectiveness of BBS resulting from their critical role in diagnosing and (re)linking people to ART, including those who have stopped being on ART 59.

Between survey rounds, programme data can indicate whether services are reaching more people and areas for improvement in programme quality. In the context of decreasing funding from external donors in countries with concentrated epidemics, it becomes ever more important to use data to inform the response and make an investment case for national and donor resources.

## Competing interests

We have no competing interests to report.

## Authors’ contributions

AH, WH, DP and KS conceived of this manuscript. AH and WH contributed substantially to the writing. VM, JZ, JB, KS, DP and JC reviewed the manuscript.
